# Quit Stage and Intervention Type Differences in the Momentary Within-Person Association Between Negative Affect and Smoking Urges

**DOI:** 10.3389/fdgth.2022.864003

**Published:** 2022-03-29

**Authors:** Lizbeth Benson, Chaelin K. Ra, Emily T. Hébert, Darla E. Kendzor, Jason A. Oliver, Summer G. Frank-Pearce, Jordan M. Neil, Michael S. Businelle

**Affiliations:** ^1^TSET Health Promotion Research Center, Stephenson Cancer Center, University of Oklahoma Health Sciences Center, Oklahoma City, OK, United States; ^2^Department of Health Promotion and Behavioral Sciences, UT Health School of Public Health, Austin, TX, United States; ^3^Department of Family and Preventive Medicine, University of Oklahoma Health Sciences Center, Oklahoma City, OK, United States; ^4^Department of Biostatistics and Epidemiology, Hudson College of Public Health, The University of Oklahoma Health Sciences Center, Oklahoma City, OK, United States

**Keywords:** digital health, mHealth, smoking cessation, just-in-time adaptive intervention (JITAI), negative affect (NA)

## Abstract

**Background:**

Smoking urges and negative affect play important roles in daily cigarette smoking and smoking lapse during a cessation attempt. Traditionally, laboratory research has considered negative affect as a potential cause of smoking urges. A deeper understanding of momentary associations between negative affect and smoking urges during a smoking cessation attempt can inform treatment development efforts. This study examined whether the within-person association between negative affect and smoking urges differed before and after a quit attempt, and by intervention type.

**Methods:**

Data are from a pilot randomized controlled trial comparing 3 smoking cessation interventions. Participants were randomly assigned to: (1) a novel, smartphone-based just-in-time adaptive intervention that tailored treatment content in real-time (Smart-T2; *n* = 24), (2) the National Cancer Institute QuitGuide app (*n* = 25), or (3) a clinic-based tobacco cessation program (TTRP; *n* = 23) that followed Clinical Practice Guidelines. All participants received up to 12 weeks of nicotine replacement therapy and completed up to 5 assessments per day (*M*_*PreQuit*_= 25.8 assessments, *SD* = 6.0; *M*_*PostQuit*_= 107.7 assessments, *SD* = 37.1) of their negative affect and smoking urges during the 7 days (*M* = 6.6 days, *SD* = 1.0) prior to their quit-date and the 29 days (*M* = 25.8 days, *SD* = 6.4) after their quit-date. Prior to analysis, repeated measures of smoking urges were decomposed into between-person and within-person components.

**Results:**

After accounting for baseline nicotine dependence, Bayesian multilevel models indicated that the extent of within-person association between negative affect and smoking urges was stronger in the post-quit stage of the intervention than the pre-quit stage. Results also indicated that in the post-quit stage of the intervention, the within-person association between negative affect and smoking urges was weaker for those in the Smart-T2 and TTRP groups compared with those in the QuitGuide group. The extent of this within-person association did not differ between those in the Smart-T2 and TTRP groups.

**Conclusions:**

These findings offer preliminary evidence that the momentary within-person association between negative affect and smoking urges increases following a quit attempt, and that the TTRP and Smart-T2 interventions may weaken this association. Research is needed to replicate and expand upon current findings in a fully powered randomized controlled trial.

**Clinical Trial Registration:**

ClinicalTrials.gov NCT02930200; https://clinicaltrials.gov/show/NCT02930200.

## Introduction

Tobacco dependence is a recurring, relapsing condition (1). While most smokers want to quit, fewer than one-third of smokers use evidence-based treatments such as medication or behavioral counseling during quit attempts (2), and most smokers require several attempts before they successfully quit (3). Measurement strategies such as ecological momentary assessment (EMA), which utilize frequent self-report about behaviors and experiences in real-time (4), have allowed researchers to better understand the complicated dynamics of thoughts, feelings, and situational contexts that smokers experience during the course of a quit attempt (5). Many EMA studies have examined momentary correlates of smoking lapse during a quit attempt, including cravings (6), proximity to other smokers (7), and recent alcohol use (8).

Smoking urges and negative affect have emerged as important predictors of smoking behaviors, as well as risk factors for lapse during smoking cessation attempts (9–14). In controlled laboratory manipulations of negative affect among dependent smokers, there is strong evidence that negative affect evokes craving to smoke (15). Similarly, the findings of research examining cue-reactivity to experimentally manipulated negative affect have indicated that negative affect is associated with more *ad libitum* smoking (16). Further, research conducted in daily life settings has found that individuals tend to experience higher negative affect on stressor days compared to non-stressor days (17, 18). This body of work has conceptualized the extent of within-person association between stress and negative affect as reactivity. In addition to more traditionally measured stressors, such as arguments with a loved one or difficulties at work, momentary increases in smoking urges may be a particularly salient daily life stressor for individuals who are trying to quit smoking. Prior research has provided some evidence of momentary within-person associations between negative affect and smoking urges (19). However, little is known about how the momentary within-person association between negative affect and smoking urges may change following the initiation of a smoking cessation attempt. In addition, it is unclear whether the strength of this association may differ based on intervention type.

Given the dynamic nature of contextual factors contributing to increased risk of smoking lapse during a quit attempt, emerging strategies such as just-in-time adaptive interventions (JITAIs), have become increasingly relevant (20). JITAIs aim to address states of vulnerability for health behaviors (such as high-risk situations) by providing support in real-time through mobile technology (21). Decision rules are used to determine when and how to provide treatment in the relevant moment. To date, most JITAIs for substance use behaviors have used decision rules that assumed predictors of substance use were time-invariant (22). Although tailored intervention content delivered in real-time has the potential to attenuate the effect of smoking urges and negative affect on the risk of smoking lapse, a deeper understanding of the momentary, quit-stage dependent, within-person association between negative affect and smoking urges is needed to further refine these decision rules.

To advance our understanding of which psychological factors may contribute to cessation-related experiences in daily life, the present study used EMA data to assess the momentary within-person association between negative affect and smoking urges in a sample of adults receiving a smoking cessation intervention. Data were analyzed using multilevel modeling to examine (1) the extent of momentary within-person association between negative affect and smoking urges during a quit attempt, (2) whether the extent of this within-person association changed from the pre-quit to the post-quit stage of the study, and (3) whether the assigned intervention type moderated the degree of this within-person association.

## Methods

### Participants

A total of 81 individuals participated in the parent study. Of these participants, 9 were excluded for lack of compliance with the study protocol (see data analysis section for minimum compliance criteria). Participants in the analytic sample (*n*_Total_ = 72; *n*_*Smart*−*T*2_= 24, *n*_*QuitGuide*_= 25, *n*_*TTRP*_= 23) were, on average, 50.17 years of age (*SD* = 11.92, Range = 23–73 years), 49% female, 64% White (24% Black or African American, 1% Native Hawaiian or Other Pacific Islander, 6% American Indian/Alaska Native, and 5% Other), and 3% Hispanic/Latino. Participants were recruited from provider or self-referrals to the Tobacco Treatment Research Program (TTRP), located at the University of Oklahoma Health Sciences Center in Oklahoma City, Oklahoma. Individuals were eligible to participate if they (1) demonstrated an English literacy level greater than the sixth grade, (2) were willing to quit smoking 7 days from their first visit, (3) were ≥18 years of age, (4) had an expired carbon monoxide (CO) level >7 ppm suggestive of current smoking, (5) were currently smoking ≥5 cigarettes per day, (6) were willing and able to attend 4 in-person assessment sessions, and (7) had no contraindications for over-the-counter nicotine replacement therapy (NRT; i.e., uncontrolled blood pressure, myocardial infarction within the past 2 weeks, or current pregnancy or plans to become pregnant during the study period). All participants provided informed consent and received compensation for their participation.

### Procedure

All study procedures were approved by the Institutional Review Board at the University of Oklahoma Health Sciences Center. The intervention procedure has been described in detail elsewhere (23). Relevant to the present study, all participants completed an in-person assessment at baseline, 7 days of EMAs pre-quit, an in-person assessment on their quit date, and an additional 29 days of EMA following the scheduled quit date. At the baseline in-person assessment, study personnel provided all participants with a smartphone (Samsung Galaxy On5) and trained them how to use the device to complete the EMA portion of the study. Participants also provided information on the typical time they woke up and went to sleep for each day of the week. This information was used to set the time of EMAs so that they were not scheduled when the participant was likely to be sleeping. On the first and last days of EMA, participants were prompted to respond to one daily diary. On days 2 through 35, participants were prompted 5 times per day, starting with a daily diary assessment ~30 min after waking and 4 subsequent random assessments, finishing no later than the participants' self-reported bedtime for the given day. Data collected on the smartphone app were deidentified and encrypted. In addition to the daily diary and random assessments, participants could also self-initiate EMAs at times when they felt an urge to smoke or times when they had already smoked.

At baseline, participants were randomized into either the (1) Smart-T2 smartphone-based smoking cessation app Smart-T2, (2) National Cancer Institute QuitGuide app, or (3) tobacco cessation clinic care (TTRP). Intervention types and study procedures have been described in detail previously (23, 24). Briefly, the Smart-T2 app featured just-in-time treatment messages delivered at the end of each EMA. Treatment messages were tailored to a participant's current risk of smoking lapse, determined by a weighted algorithm, as well as the highest rated of four momentary lapse triggers: urge to smoke, stress, cigarette availability, and low motivation to quit. The weighted algorithm was developed in a prior study that examined the optimal weight to assign to momentary responses for each of six lapse triggers (urge to smoke, stress, cigarette availability, interacting with someone smoking, recent alcohol use, and cessation motivation), by comparing the extent to which the estimator distinguished between moments of high vs. low risk for imminent smoking lapse in the next 4 hours (25). Results from that study showed that the final weighted lapse risk estimator successfully identified 80% of all first smoking lapses within 4 h of the smoking lapse. This lapse risk estimator was integrated into and informs the real-time tailoring of intervention messages for the Smart-T1 (24) and Smart-T2 JITAIs (23). The Smart-T app also featured on-demand quit tips. The NCI QuitGuide app (26, 27) is a free smartphone app available through Smokefree.gov and includes on-demand content such as the ability to track cravings, smoking triggers, and quit tips. Finally, the TTRP group received usual tobacco cessation treatment based on established clinical practice guidelines (28), including weekly individual counseling sessions. All three groups received a 2-week supply of over the counter nicotine replacement therapy (NRT; i.e., patches, gum, or both) and participants could request up to 8 additional weeks of NRT.

### Measures

Five times per day, all participants were prompted *via* smartphone to report their current experiences of negative affect, smoking urges, and smoking behaviors during the week prior to their quit date and the 4 weeks subsequent to their quit date.

#### Momentary Negative Affect

During each daily diary and random assessment, participants answered questions about their negative emotion experiences, chosen to span both high activation (i.e., stress, anxious, frustrated/angry, irritable, worried, and restless) and low activation (i.e., depressed, sad, and miserable) quadrants of the circumplex model (29). Response options for each of these questions ranged from *Strongly Disagree* (=1) to *Strongly Agree* (=5). The average within-person correlations among the negative emotion items were moderate to strong during both the pre-quit (Range = 0.31–0.56) and post-quit (Range = 0.31–0.52) stages of the study. Thus, a negative affect composite was computed as the average response to the nine discrete negative emotion items at each EMA. Additionally, we computed a high activation negative affect composite (irritable, frustrated/angry, worried, restless, anxious, stressed) and a low activation negative affect composite (sad, miserable, depressed) for each EMA to use in a set of secondary analyses (29).

#### Momentary Smoking Urge

During each daily diary and random assessment, participants reported on the extent to which they currently had an urge to smoke. Response options ranged from *Strongly Disagree* (=1) to *Strongly Agree* (=5).

#### Quit Stage

To quantify quit stage in terms of pre-quit date vs. post-quit date, each of the seven days prior to each participant's scheduled quit date were recoded as 0 and each of the 29 days on or after each participant's quit date were recoded as 1.

#### Intervention Type

Three dummy coded variables were created to indicate whether participants received the Smart-T2 intervention app (0 = no, 1 = yes), whether participants received the QuitGuide intervention app (0 = no, 1 = yes), and whether participants received the TTRP intervention (0 = no, 1 = yes). Given that there were three intervention treatment groups, only two dummy coded variables were used at a time. Three dummy coded variables were created so that, across two sets of analyses, treatment group differences across all three groups could be statistically evaluated.

#### Covariates

##### Baseline Smoking Dependence

During the baseline assessment, participants answered two items that comprise the Heaviness of Smoking Index (HSI) (30). First, participants responded to the question: *At present, how long after waking do you wait before having your first cigarette (in minutes)?*, using response options of 61+ min (=0), 31–60 min (=1), 6–30 min (=2), and ≤ 5 min (=3). Second, participants responded to the question: *How many cigarettes do you smoke per day at present?*, using response options of 1–10 cigarettes (=0), 11–20 cigarettes (=1), 21–30 cigarettes (=2), and 31+ cigarettes (=3). A smoking dependence composite was then computed as the sum of scores from the two items from each participant.

##### Momentary Smoking Status

Using all available data (daily diaries, random assessments, participant-initiated urge assessments, participant-initiated smoking assessments), a momentary “smoked yet today” variable was calculated. Specifically, for a given moment within a day, participants received a score of zero if they had not reported any smoking during that assessment or in any previous assessment that day, whereas they received a score of one if they reported smoking during that assessment or in any previous assessment that day.

##### Demographic Variables

Several demographic variables were included as covariates in secondary models including Gender (Male = 0, Female = 1), Race (0 = Non-white, 1 = White, given that the majority of the sample reported their race as White), Age (*M* = 50.2 years, *SD* = 11.8), Education (completed ≤ high school/GED = 0, at least some college = 1), and Employment (working less than full time = 0, working full time = 1).

### Data Analysis

Momentary occasions were included in the analyses if the participant provided complete data for negative affect and smoking urge. Nine participants were excluded from the analysis sample because they did not participate in both the pre-quit and post-quit stages of the study (*n* = 4), or because they did not have at least 5 measurement occasions with complete data for the main analysis variables in each stage of the study (*n* = 5). Participants excluded from the analysis sample were relatively evenly distributed across the Smart-T2 (*n* = 3), QuitGuide (*n* = 2), and TTRP (*n* = 4) treatment groups. Participants included in the analyses (*n*_*Total*_= 72) provided on average 25.8 pre-quit momentary assessments (*SD* = 6.0, Min = 5, Max = 31), and 107.7 post-quit momentary assessments (*SD* = 37.1, Min = 6, Max = 140). Altogether, 67% of the sample completed at least 75% of the prompted momentary assessments. Momentary assessments were uniformly completed across days such that, on average, participants responded to at least one assessment during 6.6 of the 7 pre-quit days (*SD* = 1.0, Min = 2, Max = 8), and 25.8 of the 29 post-quit days (*SD* = 6.4, Min = 2, Max = 29).

Data preparation steps included separating time-varying predictor variables into the between-person and within-person components (31). The time-invariant between-person component, *SmokeUrgeBP*_*i*_, was calculated as the intraindividual mean across the repeated measures of smoking urges, yielding one score per person. Similarly, the momentary within-person component, *SmokeUrgeWP*_*it*_ was calculated for each observation for each person as the deviation from their intraindividual mean (*SmokeUrgeBP*_*i*_). Prior to analysis, the between-person components for smoking urges (*SmokeUrgeBP*_*i*_) and baseline smoking dependence (*BaselineHSI*_*i*_) were sample-mean centered to facilitate interpretation with respect to the prototypical person in the sample.

#### Analysis for Research Questions 1–2

To examine (1) the extent of within-person association between negative affect and smoking urges and (2) whether quit stage moderates the extent of within-person association between negative affect and smoking urges, we fit a multilevel linear regression model,


(1) $$\begin{array}{l} NegativeAffect_{it}=\beta_{0i}+\beta_{1i}\left(SmokingUrgeWP_{it}\right)\\ \;+\beta_{2i}\left(QuitStage_{it}\right)\;+\beta_{3i}(SmokingUrgeWP_{it}\\ \;\times QuitStage_{it})+e_{it} \end{array}$$

where repeated measures of negative affect for participant *i* during moment *t* are modeled as a function of a person-specific intercept, β_0*i*_, changes driven by concurrent smoking urges, β_1*i*_, changes driven by pre- vs. post-quit stage, β_2*i*_, the interplay between smoking urges and quit stage, β_3*i*_, and residual differences, *e*_*it*_. Person-specific coefficients were simultaneously modeled as a function of person-level predictors,


(2) $$\begin{array}{r} \beta_{0i}=\gamma_{00}+\gamma_{01}\left(SmokingUrgeBP_{i}\right)+\gamma_{02}\left(BaselineHSI_{i}\right)\\ \;+\gamma_{03}\left(TxQG_{i}\right)+\gamma_{04}\left(TxTTRP_{i}\right)+u_{0i} \end{array}$$

(3) $$\begin{array}{r} \beta_{1i}=\gamma_{10}+u_{1i} \end{array}$$

(4) $$\begin{array}{r} \beta_{2i}=\gamma_{20}+u_{2i} \end{array}$$

(5) $$\begin{array}{r} \beta_{3i}=\gamma_{30}+u_{3i} \end{array}$$

where γ_00_, γ_10_, γ_20_, and γ_30_, and are sample-level parameters describing the prototypical person, and γ_01_, γ_02_, γ_03_, and γ_04_, describe how individual differences in smoking urges, baseline smoking dependence, and intervention treatment group are associated with participants' negative affect. Random effects (*u*_0*i*_*, u*_1*i*_*, u*_2*i*_*, u*_3*i*_) were allowed to co-vary with one another, but not with *e*_*it*_. Additionally, we examined whether the results held after accounting for demographic variables.

#### Analysis for Research Question 3

To examine whether intervention type moderates the within-person association between negative affect and smoking urges, we fit a multilevel linear regression model,


(6) $$\begin{array}{r} NegativeAffect_{it}=\beta_{0i}+\beta_{1i}\left(SmokingUrgeWP_{it}\right)+e_{it} \end{array}$$

where repeated measures of negative affect for participant *i* during moment *t* are modeled as a function of a person-specific intercept, β_0*i*_, changes driven by concurrent smoking urges, β_1*i*_, and residual differences, *e*_*it*_. Person-specific coefficients were simultaneously modeled as a function of person-level predictors,


(7) $$\begin{array}{r} \beta_{0i}=\gamma_{00}+\gamma_{01}\left(SmokingUrgeBP_{i}\right)+\gamma_{02}\left(BaselineHSI_{i}\right)\\ \;+\gamma_{03}\left(TxQG_{i}\right)+\gamma_{04}\left(TxTTRP_{i}\right)+u_{0i} \end{array}$$

(8) $$\begin{array}{r} \beta_{1i}=\gamma_{10}+\gamma_{11}\left(TxQG_{i}\right)+\gamma_{12}\left(TxTTRP_{i}\right)+u_{1i} \end{array}$$

where γ_00_ and γ_10_ and are sample-level parameters describing the prototypical person, and γ_01_, γ_02_, γ_03_, γ_04_, γ_11_, γ_12_, describe how individual differences in smoking urges, baseline smoking dependence, and intervention treatment group are associated with participants' negative affect and/or moderate the within-person association between smoking urges and negative affect. Random effects (*u*_0*i*_*, u*_1*i*_) were allowed to covary with one another, but not with *e*_*it*_. Additionally, we examined whether the results held after accounting for demographic variables.

For those individuals included in the analytic sample (i.e., those who had at least 5 days of complete data for each quit stage), missing data were relatively low for the pre-quit (17% across all people and all possible assessments) and post-quit (24% across all people and all possible assessments) stages. Thus, we treated missing data as missing at random. All analyses were performed in R version 4.1.1. and R Studio version 2021.09.0 (32, 33). All figures were created in R using the ggplot2 package (34). The *brms* package was used to fit the multilevel models specified above in the Bayesian statistical framework to facilitate model convergence with a full random effects structure. For each model, estimation included two chains and we specified default weakly informative prior probability distributions (35, 36). The first 1,000 samples from each chain were used for the “warm-up” phase of the sampling algorithm, and discarded. Another 8,000 samples were run in each chain after warm-up, and these 16,000 samples were used to estimate the mean for a point estimate (labeled as Estimate in the model result tables), and the 95% credible interval (labeled as 95% *CI* in the model result tables). The 95% *CI* can be interpreted as a 95% probability that the true parameter is contained in the interval.

## Results

Descriptives for and correlations among main study variables are presented in Table 1. The average intensity of momentary negative affect was similar across the pre-quit stage (*M* = 2.1 *SD* = 0.7) and the post-quit stage (*M* = 2.1 *SD* = 0.8) of the study. The average intensity of smoking urges was descriptively slightly higher during the pre-quit stage (*M* = 3.1, *SD* = 0.7) compared with the post-quit stage (*M* = 2.8, *SD* = 0.9) of the study. Given that participants were instructed to smoke as usual during the pre-quit stage, smoking rates were relatively high such that for 86% of the pre-quit moments, participants had already smoked at some point in the day. For the post-quit stage, smoking rates were much lower such that participants had already smoked at some point in the day for only 26% of the post-quit moments. Similarly, on average, participants smoked at least once per day for 80% (*SD* = 22.5, Min = 0%, Max = 100%) of their pre-quit days and smoked at least once per day for 36% (*SD* = 37.3, Min = 0%, Max = 100%) of their post-quit days. Prior to the main analyses, we calculated intraclass correlation coefficients to examine how much variance in the variables pertinent to the present study could be attributed to the momentary and person levels of analysis. For negative affect, 33% of the variation could be considered within-person variation, with the remaining 67% considered between-person variation. For smoking urges, 56% of the variation could be considered within-person variation and 44% could be considered between-person variation. Given considerable variation at both levels of analysis for negative affect (the outcome variable), we proceeded to fit the multilevel models. Results are described in the following sections and reported in Table 2.

**Table 1:** Descriptives and correlations (between- and within-person) among study variables.

		**Pre-quit date**		**Post-quit date**					
		**Negative affect**	**Smoking urge**	**Negative affect**	**Smoking urge**	**Smart-T2**	**TTRP**	**QuitGuide**	**Baseline HSI smoking dependence**
Pre-quit date	Negative affect	1.0	**0.17**	-	-	-	-	-	-
	Smoking urge	0.44	1.0	-	-	-	-	-	-
Post-quit date	Negative affect	0.80	0.51	1.0	**0.24**	-	-	-	-
	Smoking urge	0.38	0.59	0.7	1.0	-	-	-	-
	Smart-T2	−0.08	0.04	−0.02	0.18	1.0	-	-	-
	TTRP	−0.14	0.10	−0.14	−0.14	-	1.0	-	-
	QuitGuide	0.22	−0.14	0.16	−0.03	-	-	1.0	-
	Baseline HSI smoking dependence	0.21	0.13	0.31	0.34	−0.01	0.01	0.00	1.0
	Sample *Mean*	2.12	3.09	2.12	2.80	35%	32%	33%	3.56
	Sample *SD*	0.73	0.67	0.80	0.91	-	-	-	1.30

*Correlations in regular type indicate the extent of between-person association among person-level characteristics. Correlations in bold type indicate the average within-person correlation. Average within-person correlations were only calculated within a quit stage and not across quit stages given the unequal numbers of days per stage (pre-quit = 7 days, post-quit = 29 days). Smart-T2, TTRP, and QuitGuide are all binary variables indicating treatment group membership. For the pre-quit and post-quit variables, Sample Mean is the sample average of the within-person means. For the pre-quit and post-quit variables, Sample SD is the standard deviation of the sample average of the within-person means*.

**Table 2:** Results from multilevel models examining differences in within-person negative affect association with smoking urges based on quit stage.

		**Primary model**		**Model adjusting for demographic variables**	
	**Parameter**	**Est**.	**95% *CI***	**Est**.	**95% *CI***
**Fixed effects**					
Intercept	γ_00_	**2.0**	(1.8, 2.2)	**2.1**	(1.7, 2.5)
Smoking urge BP	γ_01_	**0.6**	(0.4, 0.7)	**0.6**	(0.4, 0.8)
Baseline HSI	γ_02_	0.03	(−0.08, 0.13)	0.01	(−0.11, 0.13)
TxQG	γ_03_	0.2	(−0.1, 0.5)	0.2	(−0.2, 0.5)
TxTTRP	γ_04_	0.1	(−0.3, 0.4)	0.03	(−0.31, 0.37)
Smoking urge WP	γ_10_	**0.11**	(0.07, 0.16)	**0.11**	(0.07, 0.16)
Quit stage (0 = Pre-Quit)	γ_20_	0.04	(−0.06, 0.14)	0.04	(−0.06, 0.15)
Smoking urge WP × quit stage	γ_30_	**0.06**	(0.02, 0.10)	**0.06**	(0.02, 0.10)
Female	γ_05_	-	-	−0.03	(−0.30, 0.25)
White	γ_06_	-	-	−0.07	(−0.38, 0.24)
Age	γ_07_	-	-	0.00	(−0.01, 0.01)
College	γ_08_	-	-	−0.2	(−0.5, 0.1)
Working full time	γ_09_	-	-	0.1	(−0.2, 0.4)
**Random effects**					
*SD* Intercept	σ*_*u*0*i*_*	**0.7**	(0.5, 0.8)	**0.7**	(0.6, 0.8)
*SD* Smoking urge WP	σ*_*u*1*i*_*	**0.16**	(0.12, 0.19)	**0.16**	(0.12, 0.20)
*SD* Quit stage	σ*_*u*2*i*_*	**0.42**	(0.35, 0.50)	**0.4**	(0.3, 0.5)
*SD* Smoking urge WP × quit stage	σ*_*u*3*i*_*	**0.10**	(0.05, 0.14)	**0.10**	(0.05, 0.14)
Correlation intercept, smoking urge WP	ρ(*u_0*i*_, u_1*i*_*)	−0.04	(−0.32, 0.25)	−0.06	(−0.35, 0.23)
Correlation intercept, quit stage	ρ(*u_0*i*_, u_2*i*_*)	–**0.4**	(−0.6, −0.2)	–**0.4**	(−0.6, −0.2)
Correlation intercept, smoking urge WP × quit stage	ρ(*u_0*i*_, u_3*i*_*)	**0.42**	(0.04, 0.74)	0.4	(0.0, 0.7)
Correlation smoking urge WP, quit stage	ρ(*u_1*i*_, u_2*i*_*)	0.2	(−0.1, 0.4)	0.2	(−0.1, 0.4)
Correlation smoking urge WP, smoking urge WP × quit stage	ρ(*u_1*i*_, u_3*i*_*)	−0.06	(−0.45, 0.45)	−0.1	(−0.5, 0.5)
Correlation quit stage, smoking urge WP × quit stage	ρ(*u_2*i*_, u_3*i*_*)	0.1	(−0.3, 0.4)	0.1	(−0.3, 0.4)
Residual *SD*	σ*_*ei*_*	**0.44**	(0.43, 0.45)	**0.44**	(0.43, 0.45)

*Est. = Posterior Mean; 95% CI = 95% Credible Interval; Estimates are bolded if their corresponding 95% CI does not contain zero, indicating a credible association. Data are reports on 1,858 pre-quit moments, 7,751 post-quit moments nested within 72 people*.

### The Within-Person Association Between Negative Affect and Smoking Urges Differs by Quit Stage

First, we examined whether the extent of within-person association between negative affect and smoking urges differed by quit stage (pre- vs. post-quit). The results indicated that for the prototypical participant assigned to the Smart-T2 intervention (the reference group), with average baseline smoking dependence and average momentary smoking urges, their average level negative affect was expected to be 2.0 (γ_00_) on pre-quit occasions. Further, the results showed that individuals' average level of negative affect did not credibly differ from pre-quit date to post-quit date (γ_20_ = 0.04). However, the results indicated that individuals in the sample credibly differed from one another in their average levels of negative affect during the pre-quit (σ_*u*0_ = 0.7) and post-quit (σ_*u*2_ = 0.4) stages of the study. Additionally, between-person differences in smoking urges were credibly associated with between-person differences in negative affect such that those who tended to have more intense smoking urges, on average, over the course of the study period also tended to have higher negative affect (γ_01_ = 0.6).

**Figure 1:**
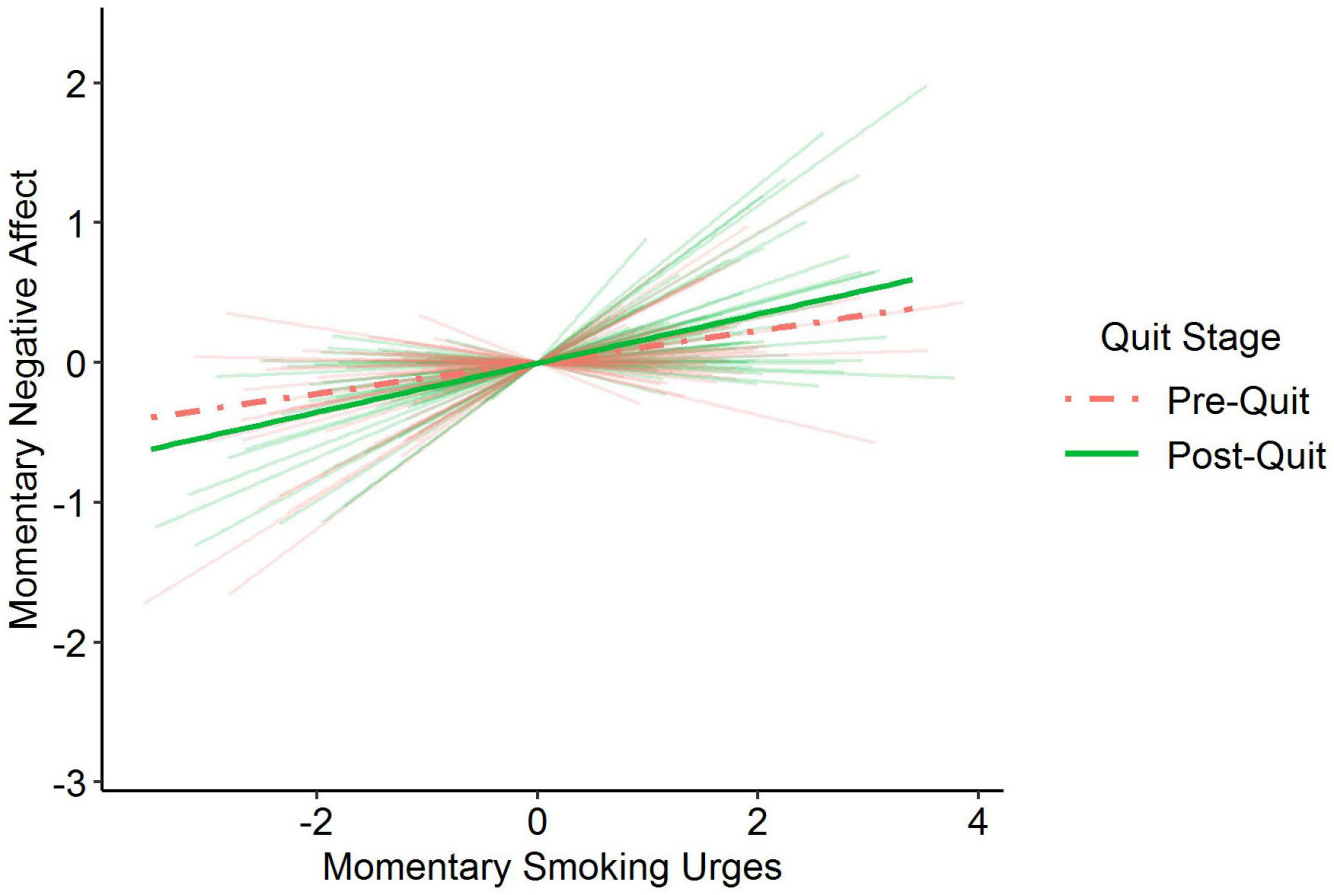
Prototypical within-person association between momentary smoking urges and momentary negative affect moderated by quit stage [pre-quit date (0 = study days 0 through 6), post-quit date (1 = study days 7 through 35)]. Individuals' negative affect and smoking urges were coupled to a greater extent during the post-quit stage (green solid line) compared with the pre-quit stage (coral dashed line). Faint background lines show the range of between-person differences in the within-person association between smoking urges and negative affect during both the pre-quit (coral) and post-quit (green) stages.

Within-person variation in smoking urges were also credibly associated with negative affect (γ_10_ = 0.1). Further, the extent of within-person association between negative affect and smoking urges was moderated by quit stage (γ_30_ = 0.1). As shown in Figure 1, there was a stronger association between smoking urges and negative affect during the post-quit stage of the intervention (green solid line) compared with the pre-quit stage of the intervention (coral dashed line). The results also showed that there was considerable heterogeneity across participants in the strength of the within-person association between negative affect and smoking urges during the pre-quit (σ_*u*1_ = 0.2) and post-quit (σ_*u*2_ = 0.1) stages (shown by the faint coral and green lines in Figure 1), and that those who tended to have higher average negative affect during the pre-quit stage also tended to have stronger links between their negative affect and their smoking urges during the post-quit stage [ρ(*u*_0*i*_, *u*_3*i*_)], but not the pre-quit stage [ρ(*u*_0*i*_, *u*_1*i*_)]. All results held after accounting for demographic covariates (see right two columns of Table 2).

### The Within-Person Association Between Negative Affect and Smoking Urges Differs by Intervention Type

**Figure 2:**
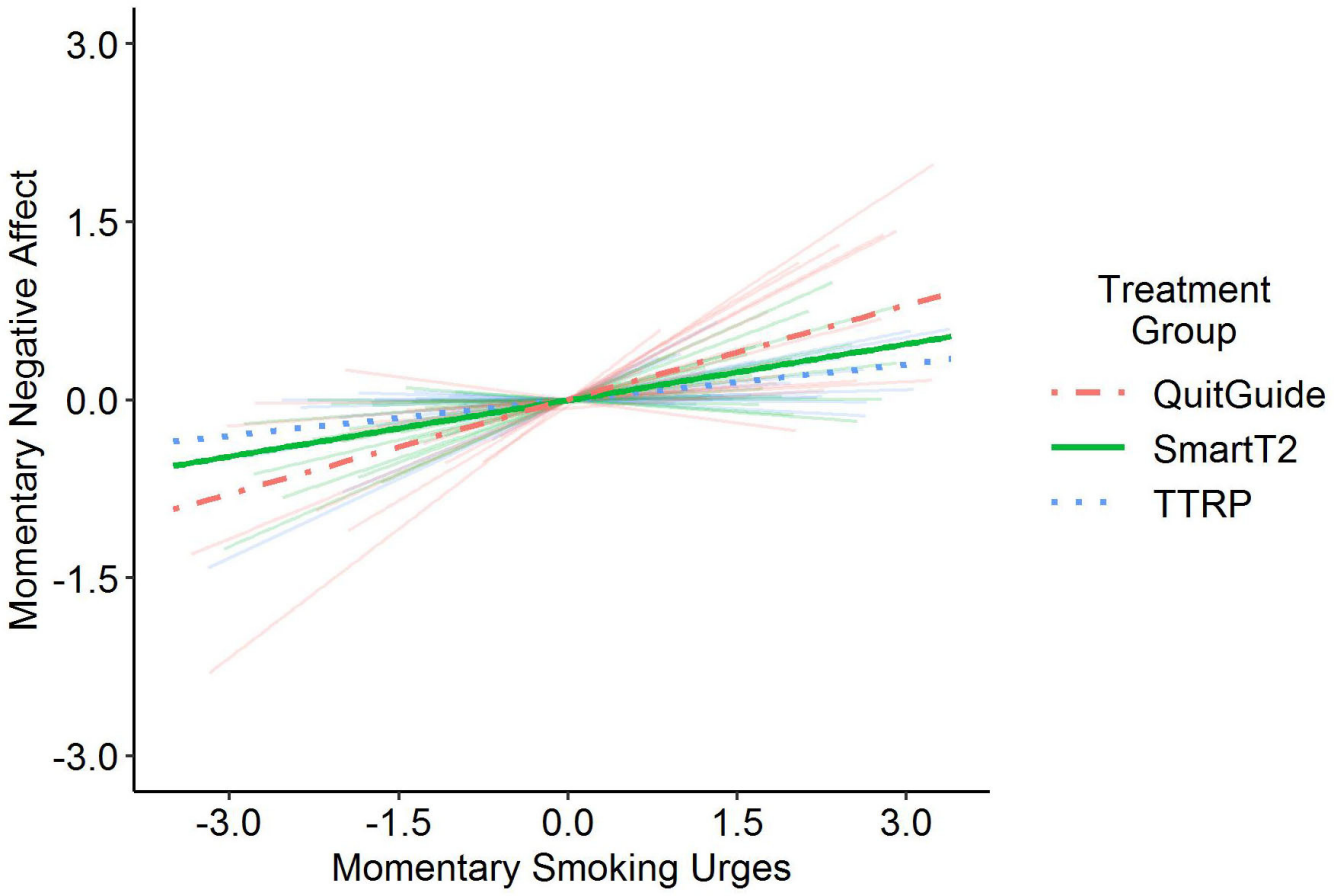
The within-person association between momentary negative affect and smoking urges was moderated by intervention type during the post-quit stage of the study. Specifically, those who received SmartT2 (green solid line) and TTRP (blue dotted line) did not differ from one another in the extent to which their momentary negative affect experiences were associated with their momentary smoking urges. Additionally, those who received QuitGuide (coral dashed line) showed a stronger within-person association between their experiences of negative affect and smoking urges compared with those who received SmartT2 and those who received TTRP. The faint background lines depict person-specific associations.

Second, we examined whether the extent of within-person association between negative affect and smoking urges differed by intervention type during the post-quit stage of the study. As shown in Figure 2, the results indicated that those who received the Smart-T2 intervention (the reference group; γ_10_ = 0.16) showed a weaker association between their momentary experiences of negative affect and smoking urges compared with those who received the QuitGuide intervention (γ_11_ = 0.1) during the post-quit stage of the study. In contrast, the extent of the within-person association between negative affect and smoking urges did not differ between those who received the Smart-T2 intervention and those who received the TTRP intervention (γ_12_ = −0.1). We also re-ran the analysis, re-specifying the TTRP group as the reference to examine whether the extent of association differed between those assigned to TTRP and those assigned to QuitGuide. The results showed that those who received the TTRP intervention showed a weaker association between their momentary experiences of negative affect and smoking urges (the reference group; γ_10_ = 0.10) compared with those who received the QuitGuide intervention (γ_11_ = 0.2). Similar to the prior section, all results held after accounting for demographic covariates (see third model in Table 3).

**Table 3:** Results from multilevel models examining differences in within-person negative affect association with smoking urges based on mHealth intervention type.

		**Primary model fit to post-quit data**		**Model fit to pre-quit data**		**Model adjusting for demographic variables**		**Model adjusting for smoking status in a given day**	
	**Parameter**	**Est**.	**95% *CI***	**Est**.	**95% *CI***	**Est**.	**95% *CI***	**Est**.	**95% *CI***
**Fixed effects**									
Intercept	γ_00_	**2.0**	(1.7, 2.2)	**2.0**	(1.8, 2.3)	**2.1**	(1.7, 2.6)	**2.0**	(1.7, 2.2)
Smoking urge BP	γ_01_	**0.6**	(0.4, 0.7)	**0.5**	(0.3, 0.7)	**0.6**	(0.4, 0.7)	**0.6**	(0.4, 0.8)
Baseline HSI	γ_02_	0.0	(−0.1, 0.1)	0.1	(0.0, 0.2)	0.0	(−0.1, 0.1)	0.0	(−0.1, 0.2)
TxQG	γ_03_	0.3	(0.0, 0.7)	**0.39**	(0.02, 0.76)	0.3	(0.0, 0.7)	0.3	(0.0, 0.7)
TxTTRP	γ_04_	0.1	(−0.3, 0.4)	−0.1	(−0.5, 0.3)	0.1	(−0.3, 0.4)	0.0	(−0.3, 0.4)
Smoking urge WP	γ_10_	**0.16**	(0.09, 0.23)	**0.13**	(0.05, 0.20)	**0.16**	(0.08, 0.23)	**0.16**	(0.09, 0.23)
Smoking urge WP × TxQG	γ_11_	**0.107**	(0.005, 0.206)	0.02	(−0.09, 0.13)	**0.107**	(0.003, 0.212)	0.102	(−0.002, 0.202)
Smoking urge WP × TxTTRP	γ_12_	−0.1	(−0.2, 0.0)	−0.06	(−0.17, 0.05)	−0.1	(−0.2, 0.1)	−0.1	(−0.2, 0.0)
Female	γ_05_	-	-	-	-	−0.1	(−0.4, 0.2)	-	-
White	γ_06_	-	-	-	-	−0.1	(−0.4, 0.2)	-	-
Age	γ_07_	-	-	-	-	0.0	(0.0, 0.0)	-	-
College	γ_08_	-	-	-	-	−0.2	(−0.5, 0.1)	-	-
Working full time	γ_09_	-	-	-	-	0.1	(−0.2, 0.4)	-	-
Proportion smoking days BP	γ_09_	-	-	-	-	-	-	–**0.6**	(−1.1, −0.1)
Smoked yet today WP	γ_20_	-	-	-	-	-	-	**0.2**	(0.1, 0.3)
**Random effects**									
*SD* Intercept	σ*_*u*0*i*_*	**0.6**	(0.5, 0.7)	**0.6**	(0.5, 0.8)	**0.6**	(0.5, 0.8)	**0.6**	(0.5, 0.7)
*SD* Smoking urge WP	σ*_*u*1*i*_*	**0.16**	(0.13, 0.20)	**0.15**	(0.12, 0.20)	**0.17**	(0.14, 0.20)	**0.16**	(0.13, 0.20)
*SD* Smoked yet today WP	σ*_*u*2*i*_*	-	-	-	-	-	-	**0.23**	(0.16, 0.30)
Correlation intercept, smoking urge WP	ρ(*u_0*i*_, u_1*i*_*)	**0.4**	(0.1, 0.6)	−0.1	(−0.4, 0.2)	**0.32**	(0.03, 0.57)	0.3	(0.0, 0.5)
Correlation intercept, smoked yet today WP	ρ(*u_0*i*_, u_2*i*_*)	-	-	-	-	-	-	0.0	(−0.3, 0.3)
Correlation smoking urge WP, smoked yet today WP	ρ(*u_1*i*_, u_2*i*_*)	-	-	-	-	-	-	0.3	(−0.1, 0.6)
Residual *SD*	σ*_ε*i*_*	**0.44**	(0.43, 0.44)	**0.45**	(0.44, 0.47)	**0.44**	(0.43, 0.44)	**0.43**	(0.42, 0.43)

*Est. = Posterior Mean; 95% CI = 95% Credible Interval; Estimates are bolded if their corresponding 95% CI does not contain zero, indicating a credible association; Data are reports on 7,751 post-quit moments nested within 72 people*.

As an additional check, we re-ran the analysis, applied to just the pre-quit data when exposure to the interventions was still relatively low and when individuals were not actively trying to quit smoking. As expected, the results showed that during the pre-quit stage of the study, there were no credible differences in the extent of within-person association between negative affect and smoking urges attributed to intervention type (γ_10_ = 0.13, γ_11_ = 0.02, γ_12_ = −0.06; second model in Table 3). For ease of presentation and interpretation, we fit the model separately to the pre-quit data and post-quit data. The results were similar though when the model was simultaneously fit to the pre- and post-quit data such that there was a credible 3-way interaction involving momentary smoking urges, intervention stage, and treatment group.

#### Sensitivity Analyses

As a sensitivity check, we fit an additional model examining whether intervention type differences in association still held after accounting for whether in a given moment the participant had smoked yet that day. Although the posterior mean estimate was similar to the primary model (γ_11_PrimaryModel_ = 0.11, γ_11_SensitivityModel_ = 0.10), the credible interval included zero for the sensitivity model. In other words, after accounting for whether a participant smoked yet in a given day, there were no longer credible differences in the extent of within-person association between negative affect and smoking urges based on whether the participant received Smart-T2 or QuitGuide. Similar to the primary model, the results showed that the within-person association between negative affect and smoking urges did not differ for those in the Smart-T2 and TTRP groups (γ_11_SensitivityModel_ = −0.07). Also similar to the primary model, when we re-fit the sensitivity analysis model with TTRP specified as the reference group, those in the QuitGuide group showed a stronger within-person association between their negative affect and smoking urges compared with those in the TTRP group during the post-quit stage (γ_11_SensitivityModel_ = 0.2).

### Secondary Analyses

In a set of secondary analyses, we examined whether the results differed based on the activation of the negative affect composites. Results are presented in Table 4.

**Table 4:** Results from secondary analyses examining negative affect association with smoking urges based on low activation and high activation negative affect.

		**Association differences Pre- vs. post-quit date**				**Association differences by intervention type**			
		**Low activation negative affect**		**High activation negative affect**		**Low activation negative affect**		**High activation negative affect**	
	**Parameter**	**Est**.	**95% *CI***	**Est**.	**95% *CI***	**Est**.	**95% *CI***	**Est**.	**95% *CI***
**Fixed effects**									
Intercept	γ_00_	**1.8**	(1.6, 2.1)	**2.1**	(1.8, 2.3)	**1.8**	(1.6, 2.1)	**2.0**	(1.8, 2.3)
Smoking urge BP	γ_01_	**0.4**	(0.2, 0.6)	**0.7**	(0.5, 0.8)	**0.4**	(0.2, 0.6)	**0.6**	(0.5, 0.8)
Baseline HSI	γ_02_	0.0	(−0.1, 0.2)	0.0	(−0.1, 0.1)	0.0	(−0.1, 0.2)	0.0	(−0.1, 0.1)
TxQG	γ_03_	0.2	(−0.2, 0.6)	0.2	(−0.1, 0.5)	0.3	(−0.1, 0.7)	0.4	(0.0, 0.7)
TxTTRP	γ_04_	0.0	(−0.4, 0.4)	0.1	(−0.2, 0.4)	0.1	(−0.3, 0.5)	0.1	(−0.3, 0.4)
Smoking urge WP	γ_10_	**0.08**	(0.03, 0.12)	**0.13**	(0.09, 0.18)	**0.08**	(0.01, 0.16)	**0.2**	(0.1, 0.3)
Smoking urge WP × TxQG	γ_11_	-	-	-	-	**0.13**	(0.02, 0.24)	0.10	(−0.01, 0.20)
Smoking urge WP × TxTTRP	γ_12_	-	-	-	-	−0.03	(−0.14, 0.08)	−0.07	(−0.18, 0.04)
Quit stage (0 = pre-quit)	γ_20_	0.07	(−0.04, 0.18)	0.03	(−0.07, 0.13)	-	-	-	-
Smoking urge WP × quit stage	γ_30_	**0.041**	(0.003, 0.079)	**0.07**	(0.03, 0.11)	-	-	-	-
**Random effects**									
*SD* Intercept	σ*_*u*0*i*_*	**0.7**	(0.6, 0.9)	**0.7**	(0.6, 0.8)	**0.7**	(0.6, 0.8)	**0.6**	(0.5, 0.7)
*SD* Smoking urge WP	σ*_*u*1*i*_*	**0.1**	(0.1, 0.2)	**0.17**	(0.13, 0.21)	**0.17**	(0.14, 0.22)	**0.17**	(0.14, 0.21)
*SD* Quit stage	σ*_*u*2*i*_*	**0.5**	(0.4, 0.6)	**0.4**	(0.3, 0.5)	-	-	-	-
*SD* Smoking urge WP × quit stage	σ*_*u*3*i*_*	**0.08**	(0.04, 0.14)	**0.11**	(0.07, 0.16)	-	-	-	-
Correlation intercept, smoking urge WP	ρ(*u_0*i*_, u_1*i*_*)	0.1	(−0.3, 0.4)	0.0	(−0.3, 0.2)	**0.31**	(0.02, 0.55)	**0.4**	(0.1, 0.6)
Correlation intercept, quit stage	ρ(*u_0*i*_, u_2*i*_*)	–**0.4**	(−0.6, −0.1)	–**0.4**	(−0.6, −0.2)	-	-	-	-
Correlation intercept, smoking urge WP × quit stage	ρ(*u_0*i*_, u_3*i*_*)	0.2	(−0.3, 0.6)	**0.5**	(0.1, 0.8)	-	-	-	-
Correlation smoking urge WP, quit stage	ρ(*u_1*i*_, u_2*i*_*)	0.2	(−0.1, 0.4)	0.2	(−0.1, 0.4)	-	-	-	-
Correlation smoking urge WP, smoking urge WP × quit stage	ρ(*u_1*i*_, u_3*i*_*)	0.3	(−0.2, 0.8)	−0.2	(−0.5, 0.3)	-	-	-	-
Correlation quit stage, smoking urge WP × quit stage	ρ(*u_2*i*_, u_3*i*_*)	0.2	(−0.2, 0.6)	0.0	(−0.3, 0.3)	-	-	-	-
Residual *SD*	σ*_ε*i*_*	**0.48**	(0.47, 0.49)	**0.48**	(0.47, 0.48)	**0.48**	(0.47, 0.49)	**0.47**	(0.47, 0.48)

*Est. = Posterior Mean; 95% CI = 95% Credible Interval; Estimates are bolded if their corresponding 95% CI does not contain zero, indicating a credible association. For the models examining differences in association based on pre- vs. post-quit date, the data are reports on 1,858 pre-quit moments, 7,751 post-quit moments nested within 72 people. For the models examining differences in association based on intervention type, the data are reports on 7,751 post-quit moments nested within 72 people*.

First, we examined whether the extent of within-person association between negative affect and smoking urges differed by quit stage using the low activation negative affect composite and the high activation negative affect composite. The results showed that for both low activation negative affect and high activation negative affect, the extent of within-person association between negative affect and smoking urges was stronger during the post-quit stage of the study compared with the pre-quit stage of the study (γ_30_ = 0.04, γ_30_ = 0.07, respectively).

Second, we examined whether the extent of within-person association between negative affect and smoking urges differed by intervention type during the post-quit stage using the low activation negative affect composite and the high activation negative affect composite. The results indicated that those who received the Smart-T2 intervention (the reference group; γ_10_ = 0.08) showed a weaker within-person association between their momentary experiences of low activation negative affect and smoking urges compared with those who received the QuitGuide intervention (γ_11_ = 0.13). Although the posterior mean estimate was similar to the prior model and the original model (γ_11_ = 0.13), there was not a credible difference between the Smart-T2 and QuitGuide groups in the extent of within-person association between high activation negative affect and smoking urges. Similar to the primary model, for both low activation negative affect and high activation negative affect, the extent of within-person association between negative affect and smoking urges did not differ between those who received the Smart-T2 intervention and those who received the TTRP intervention (γ_12_ = −0.03, γ_12_ = −0.07, respectively). Also similar to the primary model, when we re-fit the sensitivity analysis model with TTRP specified as the reference group, those who received QuitGuide group showed a stronger within-person association between their momentary experiences of negative affect and smoking urges, for both low activation negative affect and high activation negative affect during the post-quit stage (γ_11_ = 0.2, γ_11_ = 0.2, respectively).

## Discussion

The present study is the first to use EMA data to examine how within-person associations between negative affect and smoking urges change across pre-quit and post-quit stages of a smoking cessation attempt, and differ based on intervention type. Results indicated that in moments when individuals' smoking urges were higher than usual, their negative affect also tended to be higher than usual. Second, we found that the extent of within-person association between negative affect and smoking urges was stronger during the post-quit stage compared with the pre-quit stage of the study. Third, we found that the within-person association between post-quit negative affect and smoking urges did not differ for those assigned to the Smart-T2 and TTRP groups. In contrast, those who received the QuitGuide intervention showed a stronger within-person association between their momentary experiences of negative affect and smoking urges during the post-quit stage compared with the other two groups. For each of the main findings, we also observed substantial differences across participants (regardless of intervention type) in the strength of the within-person association between their momentary experiences of negative affect and smoking urges. Further, results from sensitivity and secondary analyses indicated that the moderation by intervention type may not be credible after accounting for whether a person had already smoked that day, and may be driven primarily by low activation negative emotions (i.e., sad, miserable, and depressed). Altogether, results from the present study, discussed in the following sections, contribute to a growing literature on how the relation between momentary experiences of negative affect and smoking urges in daily life may change from pre- to post-quit, and shed light on the features of interventions that may help mitigate these changes in service of smoking cessation.

### The Within-Person Association Between Negative Affect and Smoking Urges Differs by Quit Stage

Although individuals' negative affect tended to be relatively stable, on average, during the pre-quit and post-quit stages, we found evidence of substantial within-person variability in momentary negative affect during both stages. Further, we found evidence of a within-person association between momentary experiences of negative affect and smoking urges. Specifically, on occasions when individuals' negative affect was higher than usual, their smoking urges also tended to be higher than usual. This finding aligns with prior studies conducted in both laboratory and *in vivo* settings (11, 15, 16).

Extending prior work, we observed that the extent of within-person association between negative affect and smoking urges was stronger during the post-quit stage of the intervention compared with the pre-quit stage. In other words, the coupling between individuals' momentary experiences of negative affect and smoking urges tended to be stronger during times when they were actively trying to avoid smoking compared with times when they were not actively trying to avoid smoking. Prior research has shown that nicotine withdrawal can cause both urges and negative affect and that these symptoms can also increase the likelihood of smoking lapse (11, 37). Thus, it is not surprising that our findings showed that the within-person association between negative affect and smoking urges became stronger when nicotine withdrawal increased post-quit. Smoking cessation interventions that aim to de-couple the within-person covariation between negative affect and smoking urges, may reduce the salience of smoking urges, and thus help to reduce the likelihood of smoking lapse.

Study findings related to the pre-/post-quit change in the extent of within-person association between negative affect and smoking urges may have implications for future intervention development. For example, we observed that individuals who tended to have higher average negative affect during the pre-quit stage also tended to have stronger coupling between their momentary experiences of negative affect and smoking urges during the post-quit stage of the study. This finding could be applied in future interventions by using dynamic characteristics (e.g., intraindividual mean of momentary negative affect scores) calculated from pre-quit data as a screening or tailoring tool for post-quit intervention delivery. As an example, smokers who display high characteristic negative affect during the pre-quit stage (within-person mean of momentary negative affect) could receive more intervention content focused on de-coupling the association between negative affect and smoking urges than those who have lower characteristic negative affect during the pre-quit stage. Overall, these findings align with the goals of personalized medicine by highlighting potential ways to temporally and contextually tailor interventions to boost overall effectiveness (21).

### The Within-Person Association Between Negative Affect and Smoking Urge Differs by Intervention Type

Several interesting preliminary findings emerged pertaining to differences in the extent of within-person association between negative affect and smoking urges based on intervention type during the post-quit stage. First, the average within-person association between negative affect and smoking urges was stronger for those assigned to QuitGuide compared with those assigned to Smart-T2 or TTRP. One plausible explanation of this finding is that there were important differences in the extent of personalization across the interventions. For example, those assigned to TTRP received personalized feedback from a counselor on how to cope with negative affect, smoking urges, and other smoking lapse risk factors. In contrast, those assigned to Smart-T2 and QuitGuide received all intervention content *via* smartphone. It is possible that the Smart-T2 intervention partially de-coupled the momentary relationship between negative affect and smoking urges by providing tailored real-time messages focused on addressing and/or coping with these relapse risk symptoms up to 5 times per day. Relatedly, individuals in the Smart-T2 and TTRP groups both reported higher levels of treatment satisfaction compared with the QuitGuide group, and felt that their treatment “knew how to help [them] quit smoking” (23). We also checked whether this finding might be attributable to differences in intervention efficacy, but found that the 4-week follow-up abstinence rates were relatively similar across the 3 interventions (*n*_*SmartT*2_= 6 people, *n*_*QuitGuide*_= 7 people, *n*_*TTRP*_= 8 people). Together, these findings provide promising preliminary evidence that the Smart-T2 intervention may, like traditional smoking cessation counseling, reduce the extent of within-person association between momentary experiences of negative affect and smoking urges.

### High and Low Activation Negative Affect

In secondary analyses, we examined whether results differed after negative affect was parsed into high activation (e.g., frustrated/angry) and low activation (e.g., sad) components. For both high and low activation, the extent of within-person association between negative affect and smoking urges was stronger during the post-quit stage. Additionally, compared to individuals in the QuitGuide group, those in the Smart-T2 group tended to have weaker within-person association between their low-activation negative affect and their smoking urges. However, the same difference was not credibly observed for high activation negative affect. One potential explanation for this finding pertains to differences in effort required to access intervention content, and how such effort may be more or less likely invested when negative affect and/or urges are high. Participants randomized to the Smart-T2 group immediately received intervention content after completing each brief assessment and could also access intervention content on demand *via* the app. In contrast, those randomized to QuitGuide only received on-demand intervention content when they specifically sought it out by opening the QuitGuide app. The automated, prompted, and consistent delivery of the Smart-T2 intervention content may have been particularly useful in moments when individuals were experiencing higher smoking urges than usual and low activation negative affect. Prior research has shown that low activation negative affect is associated with lower approach motivation (38). In contrast, for high activation moments, it may be that the intervention differences were “washed out” because participants were in an affective state that has been shown to be associated with higher approach motivation, and thus more likely to exert effort to access (approach) intervention content from their assigned intervention (39). Future research would benefit from further study of differences in negative affect activation and effort-based differences in exposure to intervention content.

### Strengths, Limitations, and Future Directions

This study has several strengths and limitations. The dense sampling methodology that was used (i.e., up to five EMAs per day) during the 7 pre-quit days and 29 post-quit days afforded opportunities to study within-person associations between momentary smoking urges and negative affect as well as changes in the extent of within-person association by quit stage. In contrast, the present study was designed to collect pilot data on the efficacy of the Smart-T2 intervention, and thus was not specifically powered to detect between-group (e.g., intervention type) differences in the extent of within-person associations between negative affect and smoking urges. A follow-up study is currently in the data collection phase, with more individuals per intervention type, that will be better powered to detect these cross-level interactions (40).

A second limitation is that a substantial portion of the sample reported smoking on all 29 post-quit days of the study. This high rate of post-quit smoking may have implications for whether the intervention type differences in the extent within-person association between negative affect and smoking urges could be detected. Results from the sensitivity analyses suggested that on occasions when individuals had smoked at some point already that day, their negative affect tended to be higher than usual. Thus, once an individual smokes in a given day, it may be that they are less susceptible to intervention content or that intervention content may be interpreted as less relevant for the remainder of the day. Future work should focus on further disentangling the complex associations between negative affect, smoking urges, and smoking behaviors in larger, adequately powered samples.

Another limitation is that the modeling strategy used in the present study assumed consistent within-person associations during the post-quit stage; however, this may not be the case (41). As individuals progressed further into the intervention and went longer without smoking, the extent to which momentary changes in their negative affect were coupled with momentary changes in smoking urges may have diminished. Future work could benefit from implementing other modeling techniques to test whether the post-quit association parameter should be time-varying. Similarly, the present study focused on understanding concurrent within-person associations between smoking urges and negative affect, but it may also be that these associations are bidirectional. Thus, future work should examine the temporal ordering of these associations and whether the extent of within-person association in one direction is stronger than the other direction using methods such as multilevel vector autoregressive modeling (42–44). Altogether, work in this area will afford better understanding of how these within-person dynamic characteristics change over the course of smoking cessation attempt(s) and inform theory on how the smoking behavior change process operates.

### Synopsis

Findings from the present study offer preliminary evidence that the within-person association between negative affect and smoking urges increases following a quit attempt, and that the TTRP and Smart-T2 interventions may attenuate this within-person association during the post-quit stage, compared with the QuitGuide intervention. Future work should determine if these findings are replicated in a fully powered randomized controlled trial and whether interventions designed to reduce negative affect in the context of smoking urges reduce the intensity of subsequent urges as well as the likelihood of smoking lapses. Altogether, the present study adds to a growing literature that focuses on how psychological variables (e.g., urge and negative affect) covary during a quit attempt. Study findings may inform future smoking cessation JITAI development.

## Data Availability Statement

The original contributions presented in the study are included in the article/supplementary material, further inquiries can be directed to the corresponding author.

## Ethics Statement

The studies involving human participants were reviewed and approved by University of Oklahoma Health Sciences Center. The patients/participants provided their written informed consent to participate in this study.

## Author Contributions

MB and DK conceptualized the parent study and collected study data. LB conceived of the presented study idea, analyzed the data, and drafted the methods, results, tables, and figures. MB, CR, and EH provided initial feedback on the present study idea. LB, CR, and EH drafted the introduction and discussion. CR, EH, DK, JO, SF-P, JN, and MB provided conceptual feedback and edited the manuscript. All authors contributed to the article and approved the submitted version.

## Funding

This study was supported by the Oklahoma Tobacco Settlement Endowment Trust (Grant Number 092-016-0002) and used the mobile health shared resource of the Stephenson Cancer Center *via* an NCI Cancer Center Support Grant (Grant Number P30CA225520). Manuscript preparation was additionally supported by the National Institute on Drug Abuse of the National Institutes of Health under Award Number R00DA046564. This study was funded by MB's start-up package.

## Author Disclaimer

The content is solely the responsibility of the authors and does not necessarily represent the official views of the National Institutes of Health.

## Conflict of Interest

MB and DK are inventors of the Insight mHealth Platform, which was used to develop the Smart-T2 app. They receive royalties related to its use. The remaining authors declare that the research was conducted in the absence of any commercial or financial relationships that could be construed as a potential conflict of interest.

## Publisher's Note

All claims expressed in this article are solely those of the authors and do not necessarily represent those of their affiliated organizations, or those of the publisher, the editors and the reviewers. Any product that may be evaluated in this article, or claim that may be made by its manufacturer, is not guaranteed or endorsed by the publisher.
